# Versorgungsforschung in der Akutschmerztherapie: Woher kommen wir in Deutschland?

**DOI:** 10.1007/s00482-024-00845-7

**Published:** 2024-11-06

**Authors:** Nadja Nestler, Christoph Maier, Jürgen Osterbrink

**Affiliations:** 1 Institut für Pflegewissenschaft und -praxis, Paracelsus Medizinische Privatuniversität, Strubergasse 21, 5020 Salzburg, Österreich; 2 Zentrum für Public Health und Versorgungsforschung, Paracelsus Medizinische Privatuniversität, Salzburg, Österreich; 3 Universitätsklinik für Kinder- und Jugendmedizin, St. Josef-Hospital, Ruhr-Universität Bochum, Bochum, Deutschland

**Keywords:** Akutschmerztherapie, Interdisziplinäre Gesundheitsteams, Versorgungsforschung/Forschungsprojekte, Komplexe Interventionen, Schmerztherapie/Optimierungsmaßnahmen, Pain management/acute pain, Interdisciplinary health teams, Health services research/research projects, Complex interventions, Pain management/optimization measures

## Abstract

**Hintergrund:**

Bis Anfang der 2000er-Jahre wurden Optimierungsnotwendigkeiten in der Akutschmerztherapie in Krankenhäusern aufgezeigt. Dabei wurden nur wenige Erfolge in der Optimierung erreicht. Da die Akutschmerzversorgung in Deutschland anhaltend unbefriedigend blieb, wurden die ersten Versorgungsforschungsprojekte in der Schmerzmedizin initiiert. Diese sollten Verbesserungen in der Versorgung von Patientinnen und Patienten erzielen.

**Ziel:**

Zur Darstellung der Anfänge der Versorgungsforschung im Bereich der Schmerzmedizin in Deutschland werden die Forschungsprojekte „Schmerzfreies Krankenhaus“ und „Aktionsbündnis Schmerzfreie Stadt Münster“ sowie die Zertifizierungsinitiative Certkom e. V. beschrieben.

**Material und Methoden:**

Im Sinne einer komplexen Intervention erfolgten in allen dargestellten Projekten eine Ist-Analyse der Schmerzversorgung der Patientinnen und Patienten sowie des interdisziplinären Schmerzmanagements, eine Ableitung von Optimierungsmaßnahmen, deren Implementierung sowie eine nachfolgende Evaluation.

**Ergebnisse:**

Alle Projekte konnten durch eine systematische Erhebung der Ist-Situation, gezielte Planung und Einführung von Optimierungsmaßnahmen Verbesserungen in der Patientinnen- und Patientenversorgung erzielen. Entsprechende Evaluationen konnten dies darstellen.

**Schlussfolgerung:**

Durch das systematische Vorgehen in den Projekten mit Erhebung wissenschaftlicher Daten in der Versorgung wurden Optimierungen in der Akutschmerzversorgung erzielt. Der Grundstein für weitere Versorgungsforschung in der Schmerztherapie in Deutschland wurde gelegt. Jedoch offenbarten die Projekte auch Grenzen in der Einbeziehung vulnerabler Patientinnen- und Patientengruppen.

## Abstract (en)

**Background:**

Up to the early 2000s the need for optimization in the treatment of acute pain in hospitals was highlighted. Only a few successes were achieved in the optimization process. As the acute pain care in Germany remained persistently unsatisfactory, the first health services research projects in pain medicine were initiated. These were intended to achieve improvements in patient care.

**Aim:**

To illustrate the beginnings of health services research in the field of pain medicine in Germany the research projects “Pain-free hospital” and “Action alliance pain-free City of Münster” as well as the certification initiative Certkom are described.

**Material and methods:**

An analysis of the current state of pain care for patients and interdisciplinary pain management, the derivation of optimization measures, their implementation and subsequent evaluation were carried out in all the projects presented in the sense of a complex intervention.

**Results:**

All the projects could achieve improvements in patient care through a systematic survey of the current situation, targeted planning and the introduction of optimization measures. Corresponding evaluations could demonstrate these results.

**Conclusion:**

The systematic approach in the projects with the collation of scientific data in real-life care has achieved optimization in acute pain management. The foundations were laid for further healthcare research in pain treatment in Germany; however, the projects also revealed limitations in the inclusion of vulnerable patient groups.

Bereits in den 1990er-Jahren und bis Anfang der 2000er-Jahre wurden die Häufigkeit postoperativer Schmerzen, deren Behandlung und das Management auf Krankenhausstationen in Deutschland untersucht [1, 5, 22]. Häufig wurden unzureichende Behandlungen akuter postoperativer Schmerzen aufgezeigt, dies trotz zugelassener Analgetika, der seinerzeit beginnenden Einführung von Akutschmerzdiensten und der Anwendung regionaler oder rückenmarknaher Katheterverfahren oder der patientenkontrollierten Analgesie auch auf Stationen ohne besondere Überwachungsmöglichkeiten [1, 5, 22]. Schmerzen aufgrund akuter Erkrankungen in konservativen Fachdisziplinen wurden hingegen so gut wie nicht untersucht [8].

Ab Mitte der 2000er-Jahre gab es medizinische und pflegerische Leitlinien und Standards zur Akutschmerzversorgung. Diese beschrieben neben anzuwendenden Therapieverfahren und -regimen im Kontext bestimmter Operationen und/oder Behandlungen insbesondere die Notwendigkeiten zur Organisation der Akutschmerztherapie [2, 3]. Die anhaltend unzureichende Akutschmerzversorgung und lediglich graduelle Verbesserung führten damals dazu, auch in der Schmerzmedizin gezielte Forschung in der Patient*innenversorgung durchzuführen.

Ab Mitte der 2000er-Jahre gab es Leitlinien und Standards zur Akutschmerzversorgung

Ziel des vorliegenden Beitrags ist es, die Entstehung und Abfolge der Versorgungsforschungsprojekte in der Schmerzmedizin ab den 2000er-Jahren und damit ab dem Beginn der Versorgungsforschung in diesem Bereich darzustellen. Hierzu werden drei aufeinander folgende und aufeinander aufbauende Projekte der Versorgungforschung dargestellt. Das Projekt „Schmerzfreies Krankenhaus“ wurde initiiert, da eine unzureichende Akutschmerztherapie für Deutschland sowohl in der Literatur beschrieben als auch im Austausch mit Klinikern deutlich wurde [1, 22]. Dem Projekt folgte die Gründung der Gesellschaft für Qualifizierte Schmerztherapie – Certkom e. V. als Zertifizierungsgesellschaft. Die durch das Projekt „Schmerzfreies Krankenhaus“ erbrachten Optimierungen sollten hierdurch weiterverbreitet werden. Als letzter Schritt folgte dann das Projekt „Aktionsbündnis Schmerzfreie Stadt Münster“. Hier wurde über den Sektor Krankenhaus hinaus in unterschiedlichen Versorgungsbereichen das interprofessionelle Schmerzmanagement untersucht und optimiert.

## Das Projekt „Schmerzfreies Krankenhaus“

Im Jahr 2003 konnte das Projekt „Schmerzfreies Krankenhaus“ unter Kooperation der Ruhr-Universität Bochum und der Universität Witten-Herdecke als Studie mit Prä-post-Design initiiert werden. Dabei bestanden eine medizinische Leitung durch C. Maier und eine pflegewissenschaftliche Leitung durch J. Osterbrink [8]. Es war das erste interprofessionell geleitete und realisierte Forschungsprojekt zur ebensolchen Schmerzversorgung in Europa. Durch die interdisziplinäre Zusammensetzung der Forschendengruppe bot es die Möglichkeit, die unterschiedlichen Berufsgruppen hinsichtlich der notwendigen Maßnahmen zur Optimierung der Akutschmerztherapie zu adressieren. Insbesondere konnten hierdurch aber auch unterschiedliche Forschungsmethoden angewendet werden, die eine umfassende Abbildung der Versorgungsrealität erlaubten.

### Das Projekt als komplexe Intervention

**Abb. 1:**
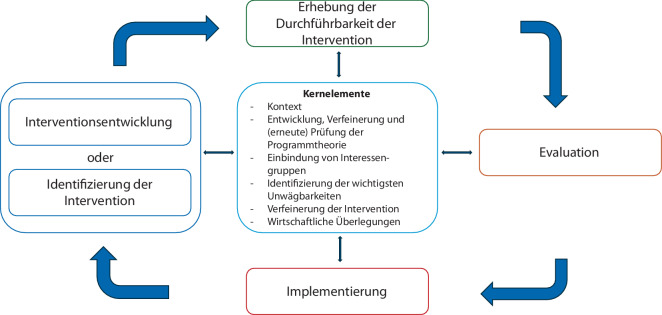
„Framework“ einer komplexen Intervention. (Eigene Darstellung, in Anlehnung an Skivington et al. 2021 [21])

Die Verbesserung der interdisziplinären Akutschmerztherapie kann als komplexe Intervention betrachtet werden (Abb. 1). Als komplexe Intervention wird eine Veränderung einer Versorgungssituation verstanden, die sich durch unterschiedliche beteiligte Berufsgruppen wie auch durch die Komplexität der umgesetzten Maßnahmen selber oder auch durch Einbeziehung unterschiedlicher Versorgungsbereiche auszeichnet [21].

Als erster Schritt einer komplexen Intervention wird die notwendige Intervention, um eine Versorgungssituation zu verbessern, identifiziert. Hierzu wird eine Ist-Analyse vorgenommen und es werden mögliche Optimierungsnotwendigkeiten abgeleitet. Nach der Entwicklung der Intervention werden die Durchführbarkeit und Akzeptanz erhoben und die geplanten Maßnahmen umgesetzt. Dieser Phase der Pilotierung folgen eine Evaluation der Zielerreichung und abschließende Implementierung im Sinne einer Ausrollung (Abb. 1). Kernelemente wie der Kontext der komplexen Intervention, die Einbindung von Interessengruppen oder die Identifizierung der wichtigsten Unwägbarkeiten sind dabei für jede komplexe Intervention leitend bzw. müssen bedacht werden.

Daher wurde im Projekt „Schmerzfreies Krankenhaus“ die Ist-Situation des interdisziplinären Akutschmerzmanagements in den teilnehmenden Kliniken erhoben. Daraus wurden Optimierungen abgeleitet und geplant. Entsprechende Optimierungsmaßnahmen wurden initiiert und nachfolgend evaluiert. Eine anschließende Adaption im Sinne der Implementierung erfolgte durch die Kliniken nach Abschluss des Projekts [7]. Die Beteiligung am Projekt muss als Pilotierung verstanden werden, da meist keine flächendeckende Optimierung in den Kliniken erfolgen konnte. Daher mussten im Sinne einer Ausrollung der Ergebnisse weitere Optimierungsschritte mit Beendigung des Projekts eingeleitet werden. Eine weitere Anpassung des interprofessionellen Schmerzmanagements erfolgte dann eigenständig durch die Kliniken. Es wurden beispielsweise Abläufe weiter optimiert oder Therapiekonzepte angepasst.

Damit wurde von Beginn an beachtet, dass die Optimierung der Akutschmerztherapie in Kliniken ein multifaktorielles Geschehen darstellt. Mit der Umsetzung von Maßnahmen müssen deren Wirkung und auch mögliche Kontextfaktoren betrachtet werden, um deren Einfluss in die Beurteilung der Optimierungsbestrebungen einbeziehen zu können (Abb. 1).

### Durchführung des Projekts

**Abb. 2:**
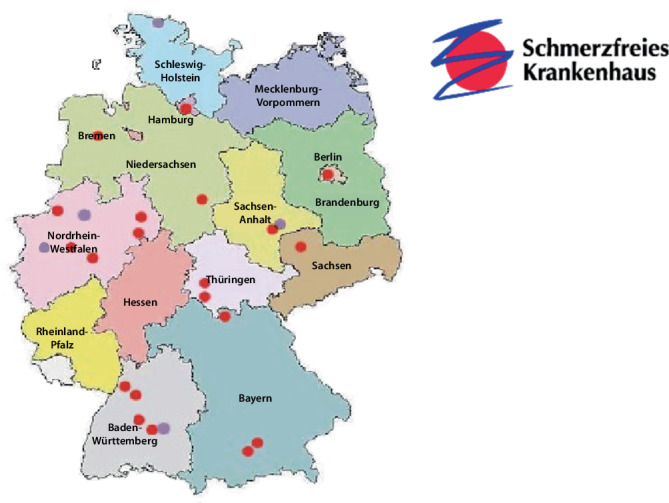
Verortung der Kliniken, die am Projekt „Schmerzfreies Krankenhaus“ teilgenommen haben

In zwei Kohorten von 5 Kliniken bzw. 20 Kliniken wurde bundesweit das interdisziplinäre Akutschmerzmanagement auf operativen und konservativen Stationen untersucht (Abb. 2). Damit gelang erstmals eine multizentrische Erhebung in Europa nicht nur in operativen, sondern auch in konservativen Abteilungen. Damit verbunden war auch das Ziel, einen ersten Einblick in die Schmerzsituation der Patientinnen und Patienten auf konservativen Stationen zu erlangen und auch ihre Schmerzversorgung zu optimieren. Somit wurde eine gezielte Umsetzung von Interventionen zur Optimierung der Akutschmerztherapie ermöglicht.

#### Zielgruppe

Im Projekt wurden operativ und konservativ behandelte Patientinnen und Patienten am ersten postoperativen Tag bzw. bis zum dritten Behandlungstag befragt. Ebenfalls wurden alle in den Bettenabteilungen tätigen Ärztinnen und Ärzte und Pflegepersonen in die Studie eingeschlossen.

#### Ablauf

In allen Kliniken erfolgte zu Beginn und nach Implementierung der Intervention eine Strukturdatenanalyse zum interdisziplinären Schmerzmanagement. Hierbei wurde die Organisation der Schmerzmedizin für die unterschiedlichen Fachbereiche und Berufsgruppen erhoben. Ebenso wurden die in den Kliniken vorhandenen Zuständigkeitsregelungen und Therapiestandards erfasst.

Eine Befragung der Patientinnen und Patienten sowie der Mitarbeitenden erfolge mittels Fragebogen. Im ersten Teilprojekt wurden zudem qualitative Forschungsmethoden angewendet und Pflegende sowie Ärztinnen und Ärzte zum Erleben der Akutschmerztherapie auf ihren Stationen bzw. in ihren Zuständigkeitsbereichen interviewt. Nichtteilnehmende Beobachtungen auf Klinikstationen aller 5 teilnehmenden Krankenhäuser wurden durchgeführt, um ein umfassendes Bild von der tatsächlichen Umsetzung des Schmerzmanagements zu erhalten.

Während der Interventionsphase wurden Ärztinnen und Ärzte sowie Pflegende zum Schmerzmanagement geschult

**Abb. 3:**
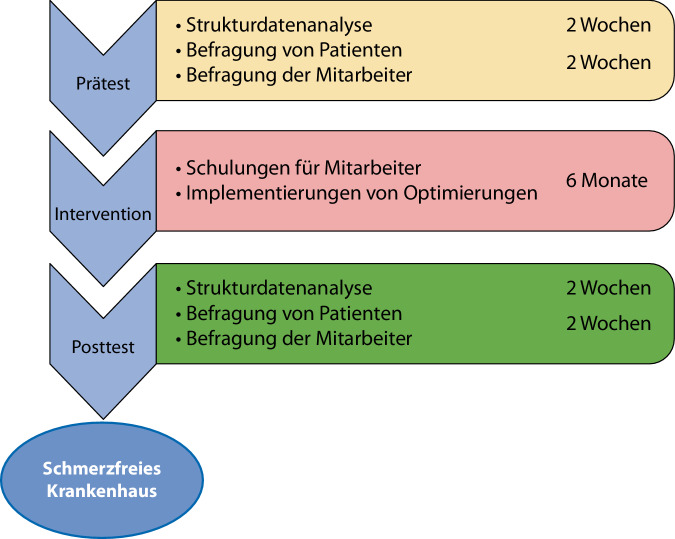
Durchführung des Projekts „Schmerzfreies Krankenhaus“

Während der Interventionsphase wurden Ärztinnen und Ärzte sowie Pflegende über einen Tag zum Schmerzmanagement geschult. Hierbei standen die Wissensvermittlung zur aktuellen Evidenz der medikamentösen wie nichtmedikamentösen Therapieoptionen sowie das interdisziplinäre Management der Akutschmerzversorgung in den verschiedenen Klinikbereichen im Fokus. Ein Nebeneffekt war die Initiierung oder auch Verbesserung der interdisziplinären Kommunikation auf Basis der durchgeführten Schulungen. Im Anschluss erfolgte die Implementierung von Optimierungen der Akutschmerzversorgung durch Anpassung oder Einführung von Therapiestandards und Festlegung von „standard operating procedures“ (SOP) für die interdisziplinäre Organisation der Schmerzversorgung im jeweiligen Klinikbereich (Abb. 3). Die Entscheidung, welche Optimierungen umgesetzt werden sollten, trafen die Kliniken auf Basis der Ergebnisse der Ist-Analyse und einer Darstellung notwendiger Optimierungen aus Sicht des Projektteams. Dies orientierte sich so weit als möglich an bestehender Evidenz.

#### Auswertung

Alle quantitativen Erhebungen wurden deskriptiv statistisch ausgewertet. Die Interviewdaten und Daten der nichtteilnehmenden Beobachtung wurden mit der qualitativen Inhaltsanalyse nach Mayring ausgewertet [9].

### Ergebnisse des Projekts

Es wurden 1282 Ärztinnen und Ärzte (Anästhesistinnen/Anästhesisten, Operateurinnen/Operateure, Ärztinnen/Ärzte konservativer Bettenstationen) und 2840 Pflegende der operativen und konservativen Abteilungen zur Akutschmerzversorgung im jeweiligen Bereich befragt [8]. Zudem wurden 4156 operativ und 1704 konservativ versorgte Patientinnen und Patienten zu ihrer Schmerzsituation am ersten postoperativen Tag bzw. bis zum dritten Behandlungstag auf konservativen Stationen befragt. Aus allen Kliniken konnten Strukturdaten zur Organisation der interprofessionellen Schmerzversorgung, zu Therapiestandards und zu Zuständigkeitsregelungen erhoben werden. Allerdings zeigten die Ergebnisse häufig ein Fehlen eindeutiger Regelungen und unklare Organisationsstrukturen für die Umsetzung und Aufgabenteilung der interprofessionellen Schmerzversorgung.

Die aus den Ist-Erhebungen abgeleiteten Ergebnisse zeigten Optimierungsnotwendigkeiten in der Schmerzversorgung von Patientinnen und Patienten nach Operationen bzw. mit akuten Erkrankungen [8]. Während der Ruheschmerz meist gut behandelt war, zeigten über 50 % aller Patientinnen und Patienten einen zu hohen und für sie nicht tolerablen Belastungsschmerz. Auch gab mehr als die Hälfte der Patientinnen und Patienten der Schmerztherapie eine unzureichende Note. Schmerzspitzen traten zumeist außerhalb der Regelarbeitszeiten am späten Nachmittag oder in der Nacht auf [8].

Seitens der Mitarbeitenden bestand häufig Unklarheit über das Vorhandensein von Zuständigkeitsregelungen für die Schmerztherapie oder auch von Therapiestandards [12]. Die Qualität der Schmerztherapie schätzten die unterschiedlichen Mitarbeitendengruppen different ein. Hier unterschied sich häufig die Wahrnehmung der Ärztinnen und Ärzte von der der Pflegenden für die gleichen Versorgungsbereiche, wobei die Einschätzungen der Pflegenden deutlich kritischer ausfielen. Die Ärztinnen und Ärzte schätzten die Qualität der Schmerztherapie häufig besser ein und waren zufriedener mit der interprofessionellen Kommunikation als die Pflegenden. Diese sahen häufiger einen Veränderungsbedarf.

Pflegende schätzten die Qualität der Schmerztherapie deutlich kritischer ein als Ärztinnen und Ärzte

Nach der Intervention konnte über alle Kliniken eine Verbesserung der Schmerzversorgung der Patientinnen und Patienten erzielt werden. Es wurden Zuständigkeitsregelungen für einzelne Aufgaben und Verantwortlichkeiten im Akutschmerzversorgungsprozess für die verschiedenen Berufsgruppen geschaffen. Therapiestandards wurden in den Kliniken festgelegt und umgesetzt. Auch das Wissen und die Einschätzung der Qualität der Schmerztherapie durch die Mitarbeitenden verbesserte sich nach der Intervention, war hier aber abhängig vom Grad der Strukturierung der Akutschmerzversorgung [13]. Die Mitarbeitenden bewerteten die Qualität der Schmerztherapie umso besser, je eindeutiger die Schmerzversorgung durch Festlegung von Verantwortlichkeiten und Zuständigkeiten geregelt war.

**Abb. 4:**
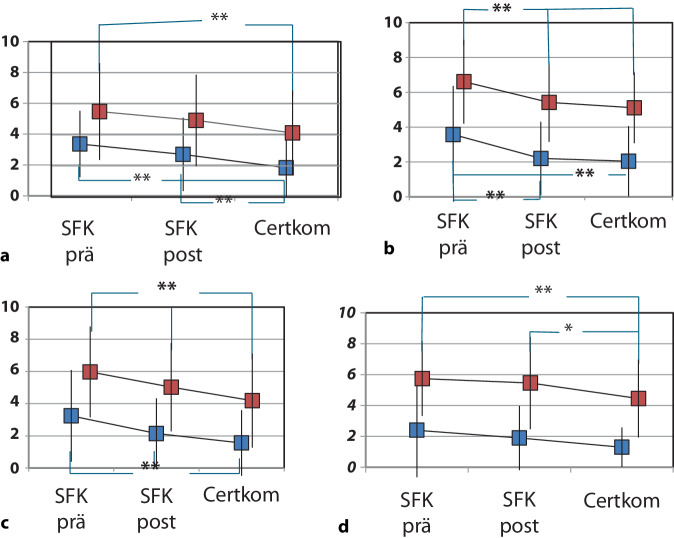
Prä-post-Vergleich der Schmerzintensität in Ruhe (*blau*) und unter Belastung (*rot*), erhoben mit NRS (0 = kein Schmerz bis 10 = stärkster vorstellbarer Schmerz). **a** Naseneingriff, **b** Bronchial Ca/Lungenmetastasen, **c** nicht-maligne gastrointestinale Erkrankungen, **d** Thorakotomie; *NRS* numerische Ratingskala, *SFK prä* „Schmerzfreies Krankenhaus“, Erhebung vor der Intervention, das heißt in der ersten Befragung und vor der Edukation, *SFK post* „Schmerzfreies Krankenhaus“, Erhebung nach der Intervention in 25 Kliniken, *Certkom* Erhebung im Rahmen der Zertifizierungsprozesse in 18 Kliniken. *Doppelstern* Signifikante Verbesserung im Vergleich zur vorherigen Periode

Mit verbindlichen Handlungsoptionen in Form von festgelegten Assessmentverfahren, Therapiestandards und Zuständigkeitsregelungen konnten die Schmerzen der Patientinnen und Patienten gesenkt werden, insbesondere der Belastungsschmerz. Dies zeigt Abb. 4 beispielhaft für einzelne Eingriffe (Naseneingriff bzw. Thorakotomie) und Erkrankungen (Bronchialkarzinom, nichtmaligne gastrointestinale Erkrankungen). Bei beiden Eingriffen sowie bei den konservativ behandelten Erkrankungen sank die Intensität der Schmerzen in Ruhe und auch unter Belastung teilweise signifikant. Die ebenfalls hier gezeigten Ergebnisse aus dem nachfolgenden Zertifizierungsprojekt (Certkom) dokumentieren bemerkenswerte mittlere Absolutwerte mit Werten auf der numerischen Ratingskala (0–10) < 3 in Ruhe bzw. < 6 unter Belastung, selbst bei in der Regel hoch schmerzhaften Eingriffen oder fortgeschrittener Metastasierung.

### Schlussfolgerungen

Damit zeigte die Ist-Erhebung im Projekt „Schmerzfreies Krankenhaus“ ähnliche Ergebnisse, wie bereits aus der internationalen Literatur für die Akutschmerzversorgung bekannt [4]. Es konnte eine deutliche Verbesserung der Schmerzsituation der Patientinnen und Patienten auf operativen wie auch auf konservativen Stationen durch Etablierung von Strukturen und Prozessen für alle beteiligten Berufsgruppen erreicht werden, insbesondere für die Pflegefachpersonen sowie Ärztinnen und Ärzte.

## Gesellschaft für Qualifizierte Schmerztherapie – Certkom e. V. als Konsequenz

Mit Beendigung des Projekts „Schmerzfreies Krankenhaus“ bestand der Wunsch der Kliniken, die erreichten Verbesserungen für die Patientinnen- und Patientenversorgung transparent nach außen darstellen zu können. So wurde von drei medizinischen Fachgesellschaften und dem pflegerischen Berufsverband die Gesellschaft Certkom e. V. mit dem Ziel der Zertifizierung der Akutschmerztherapie gegründet. Beteiligt waren:

- Deutsche Schmerzgesellschaft e. V., damals Deutsche Gesellschaft zum Studium des Schmerzes e. V.
- Deutsche Gesellschaft für Palliativmedizin e. V.
- MEDICA Deutsche Gesellschaft für Interdisziplinäre Medizin e. V.
- Deutscher Berufsverband für Pflegeberufe e. V.

Damit war auch das Ziel verbunden, die Akutschmerzversorgung in der Fläche sowohl für operative als auch für konservative Fachbereiche weiterzuentwickeln.

### Vorgehen im Zertifizierungsprozess

Kliniken mussten für die Zertifizierung neben Strukturen wie Zuständigkeitsregelungen und Therapiestandards sowie Prozessen auch Ergebnisse in der Schmerzversorgung nachweisen. Diese wurden über Patientinnen- und Patientenbefragungen erhoben. Neben der Erfassung des Ruhe‑, Belastungs- sowie Maximalschmerzes wurden auch mögliche Mobilitätseinschränkungen infolge von Schmerzen und besonders schmerzhaft erlebte Situationen erfasst. Darüber hinaus bewerteten Patientinnen und Patienten die Qualität der erlebten Schmerztherapie mit Schulnoten. Sie gaben Auskunft zur Wirksamkeit der erhaltenen Schmerztherapie sowie über mögliche Wartezeiten bis zum Erhalt eines Schmerzmedikaments [14].

Alle Ergebnisse wurden mit Grenzwerten abgeglichen, die mit Daten des Projekts „Schmerzfreies Krankenhaus“ und später mit Daten von Certkom e. V. festgelegt wurden. Auf dieser Basis wurde dann über die Zertifizierbarkeit einer Klinik entschieden. Die über die Zertifizierungserhebungen erfassten Daten stammten aus der Alltagsversorgung der Kliniken. Im Laufe der Jahre konnte so eine stetige Verbesserung der Akutschmerzversorgung der Patientinnen und Patienten in operativen wie konservativen Fachabteilungen aufgezeigt werden (Abb. 4; [7]).

## Aktionsbündnis Schmerzfreie Stadt Münster

Nach Etablierung national anerkannter Strukturen und Prozesse für eine optimierte Akutschmerztherapie in Kliniken über die Zertifizierungsverfahren von Certkom e. V. konnte die Schmerzversorgung in unterschiedlichen Versorgungsbereichen durch das Forschungsprojekt „Aktionsbündnis Schmerzfreie Stadt Münster“ untersucht und optimiert werden [16]. Die wissenschaftliche Projektgruppe untersuchte von 2010 bis 2015 in

**Abb. 5:**
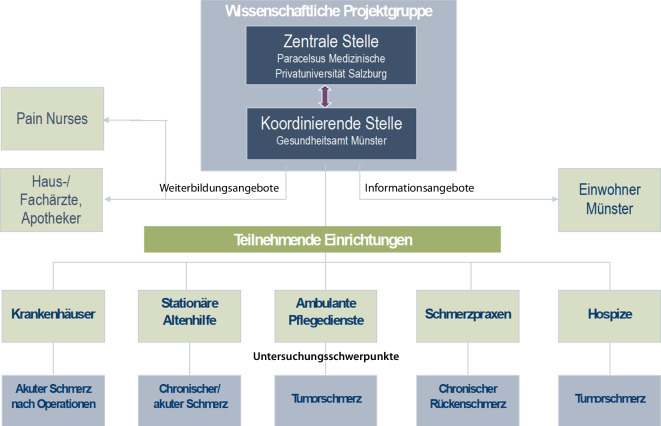
Struktur des Projekts „Aktionsbündnis Schmerzfreie Stadt Münster“

- Krankenhäusern,
- Einrichtungen der stationären Altenhilfe,
- ambulanten Pflegediensten,
- Schmerzpraxen und
- Hospizen

in der Stadt Münster das interdisziplinäre Schmerzmanagement (Abb. 5). Gemeinsam mit den Einrichtungen wurden Interventionen zur Optimierung des jeweiligen Schmerzmanagements eingeführt und zum Abschluss mögliche Veränderungen erhoben.

### Vorgehen im Projekt

In allen Einrichtungsarten wurden sowohl Mitarbeitende als auch Patientinnen und Patienten befragt. In den Einrichtungen der stationären Altenhilfe erfolgten zudem Fremdeinschätzungen des Schmerzes bei Bewohnerinnen und Bewohnern, die nicht zur Selbstauskunft über ihre Schmerzsituation in der Lage waren.

### Ergebnisse des Projekts

In allen Versorgungsbereichen konnten Optimierungsnotwendigkeiten erhoben werden, die in die jeweiligen Einrichtungen rückgespiegelt wurden. In den teilnehmenden Krankenhäusern, den Einrichtungen der stationären Altenhilfe und den ambulanten Pflegediensten wurden Schulungen zum Schmerzmanagement durchgeführt. Optimierungen des interdisziplinären Schmerzmanagements in den Kliniken sowie des pflegerischen Schmerzmanagements in den Langzeitpflegebereichen wurden implementiert.

Die abschließende Erhebung zeigte auch in diesem Projekt eine deutliche Verbesserung der Schmerzversorgung der jeweiligen Patientinnen- und Patientengruppen auf [15, 17, 19]. Allerdings waren Versorgungsbrüche zwischen den Sektoren und damit Einrichtungen immanent gegeben.

Durch öffentlichkeitswirksame Veranstaltungen wie Bürgertelefone und Bürgerveranstaltungen zu unterschiedlichen Schmerzthemen konnten Dynamiken in der Stadt mit gut 300.000 Einwohnern hinsichtlich des Themas der Schmerzversorgung in verschiedenen Versorgungsbereichen geschaffen werden. Hierdurch wurde eine nachhaltige Verortung des Themas in der Stadt ermöglicht. Versorgungsforschung konnte nicht nur Versorgungsdefizite und Optimierungsnotwendigkeiten aufzeigen sowie Verbesserungsmaßnahmen implementieren und evaluieren, es wurde auch über das Projekt hinaus das Bewusstsein in der Bevölkerung gestärkt.

## Kritische Reflexion

Im Nachgang soll eine kritische Reflexion dieser ersten Versorgungsforschungsprojekte in der Schmerzmedizin erfolgen. Die hier beschriebenen Projekte zeigen Besonderheiten der Versorgungs- oder Implementierungsforschung. Es wird in der realen Versorgung geforscht, sodass diese Versorgungsrealität auch Einfluss auf die Umsetzung der geplanten Forschung nimmt. Damit ist gegebenenfalls das Vorgehen nicht wie geplant möglich oder die gewünschte Population nur bedingt zu erreichen. Damit zeigen diese Projekte bereits auf, was heute diskutiert wird. Es werden Wirkmodelle und Monitoring sowie Struktur- und Prozessevaluationen benötigt, um bei Auffälligkeiten im Prozess frühzeitig eingreifen zu können oder diese zu beschreiben und Ergebnisse hierdurch erklären zu können (s. Beiträge von Gnass et al., Berger et al. in dieser Ausgabe). Es zeigt sich auch, dass grundlegende theoretische Modelle wie das einer komplexen Intervention gebraucht werden [21]. Diese können eine Struktur für die Planung und Umsetzung komplexer Versorgungsforschungsprojekte bieten und damit eine Orientierung für die Forschenden sein.

In den dargestellten Projekten gab es einige Herausforderungen aus dem Versorgungsalltag, die auf die Umsetzung der geplanten Forschung Auswirkungen hatten. Diese werden abschließend reflektiert. Sowohl im Projekt „Schmerzfreies Krankenhaus“ als auch im Projekt „Aktionsbündnis Schmerzfreie Stadt Münster“ offenbarten die Erhebungen nicht nur Optimierungsnotwendigkeiten in der Schmerzversorgung von Patientinnen und Patienten mit akuten Schmerzen in Deutschland. Es zeigten sich auch Barrieren in der Erhebung der Daten. Körperliche Einschränkungen aufgrund einer erfolgten Operation oder akuten Erkrankung führten zu einer höheren Quote interviewergeleiteter Befragungen als primär geplant. Patientinnen- und patientenbedingte Einschränkungen bei der Datenerhebung müssen daher mitgedacht werden und beispielsweise durch den Einsatz von Studienpersonal kompensiert werden. Es hat sich aber gezeigt, dass auch in klinischen Erhebungen Patientinnen- und Patientengruppen, die aufgrund körperlicher oder kognitiver Einschränkungen als vulnerabel zu bezeichnen sind, zumindest meist unterrepräsentiert sind.

Im Versorgungsforschungsprojekt „Aktionsbündnis Schmerzfreie Stadt Münster“ konnten weitere Forschungsproblematiken identifiziert werden. So waren in den Einrichtungen der stationären Altenhilfe, aber auch über die ambulanten Pflegedienste vulnerable Gruppen inkludiert. Es zeigten sich forschungsethische und rechtliche Probleme bei der Rekrutierung dieser Patientinnen- und Patientengruppen [20]. So musste ein erheblicher Mehraufwand für die Rekrutierung kognitiv eingeschränkter Personen in der stationären Altenhilfe geleistet werden, indem das Einverständnis der gesetzlichen Betreuenden eingeholt wurde [18].

Ebenfalls im Projekt „Aktionsbündnis Schmerzfreie Stadt Münster“ zeigten sich in der Versorgung durch ambulante Pflegedienste Rekrutierungsprobleme aufgrund des vorab festgelegten Einschlusskriteriums der Versorgung durch einen ambulanten Pflegedienst [17]. Hier präsentierte sich die Notwendigkeit einer guten Kenntnis der Versorgungsbedingungen, um sinnvolle Einschlusskriterien zu bestimmen. Hinzu kamen in diesem Projekt ethische Bedenken der zuständigen Ethikkommission bezüglich der Befragung von Hospizgästen, denen begegnet werden musste. Hierbei ist auch seitens der Forschenden eine Offenheit gegenüber möglichen Risikoeinschätzungen notwendig.

Auch muss dargestellt werden, dass sowohl die Forschungsprojekte „Schmerzfreies Krankenhaus“ und „Aktionsbündnis Schmerzfreie Stadt Münster“ als auch die Zertifizierungsgesellschaft Certkom e. V. Unterstützung durch Industriemittel erhalten haben. Ohne eine solche finanzielle Unterstützung wären die obigen Aktivitäten allerdings nicht möglich gewesen. Seinerzeit existierten keine kompetitiven Antragsmöglichkeiten und somit Mittel für Versorgungsforschung. Die Unterstützung der Projekte wurde transparent kommuniziert und vertraglich geregelt. Eine inhaltliche Einflussnahme war nicht gegeben. Sowohl die Forschungsteams als auch das Team von Certkom e. V. waren in der Planung und Durchführung der jeweiligen Projekte und Zertifizierungsverfahren unabhängig.

Alle Projekte waren Meilensteine in der interprofessionellen Versorgungsforschung der Schmerzmedizin in Deutschland. Sie waren für weitere Forschung der darauf folgenden Dekade leitend, nicht zuletzt für Projekte, die über den Innovationsfonds des Gemeinsamen Bundesausschusses bewilligt wurden.

Insbesondere haben alle hier dargestellten Projekte die Notwendigkeit einer systematischen Untersuchung der Akutschmerzversorgung dargestellt. Dies wird auch im HTA-Bericht 126 „Akutschmerztherapie auf operativen und konservativen Stationen“ aufgegriffen ([5]; *HTA* „health technology assessment“). Auch auf nationale und internationale Benchmark-Projekte hatten die Projekte Einfluss. Das eigene Vorgehen wurde durch die vorausgehenden Methodiken der Erhebungen sowie die gewonnenen Erkenntnisse beeinflusst [10, 11, 23].

## Fazit für die Praxis

- Die Optimierung der Akutschmerztherapie in Kliniken ist ein multifaktorielles Geschehen.
- Im Projekt „Schmerzfreies Krankenhaus“ konnte eine deutliche Verbesserung der Schmerzsituation der Patientinnen und Patienten auf operativen wie auch auf konservativen Stationen durch Etablierung von Strukturen und Prozessen für alle beteiligten Berufsgruppen erreicht werden.
- Das Projekt Certkom e. V. konnte im Laufe der Jahre eine stetige Verbesserung der Akutschmerzversorgung in operativen wie konservativen Fachabteilungen aufzeigen.
- Das „Aktionsbündnis Schmerzfreie Stadt Münster“ konnte nicht nur Versorgungsdefizite und Optimierungsnotwendigkeiten aufzeigen sowie Verbesserungsmaßnahmen implementieren und evaluieren, es wurde auch das Bewusstsein in der Bevölkerung hinsichtlich der Schmerzversorgung gestärkt.
- Alle Projekte waren Meilensteine in der interprofessionellen Versorgungsforschung der Schmerzmedizin in Deutschland. Insbesondere haben alle hier dargestellten Projekte die Notwendigkeit einer systematischen Untersuchung der Akutschmerzversorgung aufgezeigt.
